# Can context justify an ethical double standard for clinical research in developing countries?

**DOI:** 10.1186/1744-8603-1-11

**Published:** 2005-07-26

**Authors:** Megan Landes

**Affiliations:** 1 London School of Hygiene and Tropical Medicine, 1 Keppel Street, London, WC1E 7HT, UK

## Abstract

**Background:**

The design of clinical research deserves special caution so as to safeguard the rights of participating individuals. While the international community has agreed on ethical standards for the design of research, these frameworks still remain open to interpretation, revision and debate. Recently a breach in the consensus of how to apply these ethical standards to research in developing countries has occurred, notably beginning with the 1994 placebo-controlled trials to reduce maternal to child transmission of HIV-1 in Africa, Asia and the Caribbean. The design of these trials sparked intense debate with the inclusion of a placebo-control group despite the existence of a 'gold standard' and trial supporters grounded their justifications of the trial design on the context of scarcity in resource-poor settings.

**Discussion:**

These 'contextual' apologetics are arguably an ethical loophole inherent in current bioethical methodology. However, this convenient appropriation of 'contextual' analysis simply fails to acknowledge the underpinnings of feminist ethical analysis upon which it must stand. A more rigorous analysis of the political, social, and economic structures pertaining to the global context of developing countries reveals that the bioethical principles of beneficence and justice fail to be met in this trial design.

**Conclusion:**

Within this broader, and theoretically necessary, understanding of context, it becomes impossible to justify an ethical double standard for research in developing countries.

## Introduction

The design of clinical research trials deserves special caution, for such research is always at risk of crossing the fine line between regard for individual rights and potential exploitation of research subjects. Infamous experiments like the Tuskagee Syphilis Study have rendered evident the dangers for individuals when we cross that line. To safeguard human subjects, the international community has agreed on standard ethical principles, particularly the World Medical Association's Declaration of Helsinki; while it is encouraging that these frameworks exist, they remain open to interpretation, revision, and debate. With the 1994 placebo-controlled trials to reduce maternal to child transmission (MTCT) of HIV-1 initiated in Africa, Asia, and the Caribbean, we saw a breach in our consensus concerning the application of these principles, namely how to apply ethical standards to research conducted in the context of resource-poor settings.

In fact, the 'contextual' apologetics for this breach are inherent, I will argue, in current bioethical methodology. As an application of ethical theory, bioethics pays particular attention to context by acknowledging the unique influence of relationships and the immediate environment on an individual's experience. In terms of developing countries, bioethics has grounded its contextual analysis on the discourse of scarcity and sacrifice [1]. In particular, the use of placebo-controlled trials in developing countries has been justified by the contextual considerations of scarcity – trials that would otherwise be deemed unethical in developed countries. This convenient appropriation of 'contextual' analysis simply fails to acknowledge the underpinnings of feminist ethical analysis upon which it must stand. A more rigorous consideration of the political, social, and economic structures pertaining to individual contexts must be sought, and we must ensure that our international ethical standards are scrutinized and applied at the appropriate level. Within this broader, and theoretically necessary, understanding of context, it becomes impossible to justify an ethical double standard for research in developing countries.

## Background: The debate over placebo-controlled trials in developing countries

The debate over the application of research ethics in developing countries surfaced with the early prevention of MTCT of HIV trials. While in 1994 there was an existing protocol from the AIDS Clinical Trial Group 076 (ACTG 076) for preventing MTCT, high antiretroviral (ARV) costs and insufficient infrastructure placed the regimen out of reach for the majority of the HIV-infected population in the developing world. To find a more cost-effective and applicable treatment for resource-poor settings, randomized placebo-controlled trials were initiated to investigate a short-course ARV regimen. However, these studies sparked intense debate as they clearly violated the condition of equipoise: that placebo groups are only deemed ethical if there exists sufficient uncertainty regarding the merit of the intervention. In other words, if there exists no gold standard of care then placebos can be justified. The arguments for not providing the 'gold standard' available in developed countries were founded on the existing low 'standard of care' in the context of developing countries. The NIH and CDC, both principal funding organizations of the studies, defended the studies' design: "it is an unfortunate fact that the current standard of perinatal care for the HIV-infected pregnant women in the sites of the studies does not include any HIV prophylactic intervention at all" and that placebo controls "will be the most reliable answer to the question of the value of the study compared to the local standard of care [2]." In opposition, Paul Lurie, wrote to the United States Department of Human Rights and Services: "unless you act now as many as 1002 newborn infants will die of unnecessary HIV infections they will contract from...HIV-infected mothers in nine unethical research experiments funded by your Department [3]." Despite this early opposition, the trials began in 16 countries and included over 12 000 HIV-infected women [4].

## Redefining the 'context' of developing countries

While traditional ethical theory seeks fundamental principles to guide our actions, much of the current bioethical literature rejects claims to the effect that morality can be reduced to a set of universal principles [5]. They argue that the agent of moral decision (a patient, family member or physician) is inextricably embedded in a complex web of relationships. By accounting for the uniqueness of each ethical situation, bioethics attempts to apply ethical theory or principles relevantly to the context at hand. The problem then becomes how to define context, for much of our practical application of bioethics hinges on this point. A specifically feminist bioethics also rejects the universal claims of traditional ethical theory, but goes further to place value on the political, economic, and social factors that differentiate individual situations. In doing so, it pays particular attention to power differentials that exist between men and women, rich and poor, developed and developing countries [6]. It is this broader scope of context that must be applied to research standards in the debate over the short-course ARV placebo-controlled trials.

## Applying the principles of bioethics at an appropriate level of analysis

Since the Belmont Report defined the four principles of bioethics, namely the principles of non-maleficence, beneficence, justice and autonomy, they have been used to ensure that ethical standards are applied to research. To begin, the principle of non-maleficence states that research must cause no harm to subjects and the principle of beneficence states that due to their participation in research, all possible benefits to subjects should be maximized. This immediately raises some questions for the case at hand. While it can be argued that no outright harm was afforded to the mother-infant pairs in the placebo group since their access to ARVs was no different than it would have been within their country context, it raises the question of to whose standard of care was the trial responsible? Moreover, can we further justify using this low standard of care within a resource-rich, developed world led research trial, thus violating the principle of beneficence? What is missing is an acknowledgement of the interlocking political, social, and economic contextual factors of these trials and an examination of what 'standard of care' ought to mean. Ultimately, the question becomes whether the contextual argument is enough to justify violating the principle of beneficence.

To answer this question, we must first make a distinction between the accessibility of AZT within the developing country as opposed to accessibility in a clinical trial. Supporters of the placebo-control design did not argue that it was financially or logistically unfeasible to provide the gold standard ACTG076 regimen for the control group, but rather that it was unnecessary because of the low standard of care which existed in the developing countries. Lurie and Wolfe argue that this contextualized 'standard of care' justifies withholding a readily available treatment [7]. An individual's right to receive maximum benefits from participating in research is thus determined by their individual socio-economic status, and in turn, their access to health care.

The tenuous nature of this contextualized justification becomes clear when we take a broader view of the factors determining an individual's access to health care. First, we must acknowledge that a person's scope of choice is often determined by forces beyond her own control. As Amartya Sen describes, not only do those in developing countries face economic deprivations, they are in turn subject to substantial 'unfreedoms'. These 'unfreedoms' include lack of employment (or freedom to participate in the market) and lack of access to health care (or freedom to ward off early mortality) [8]. In particular, access to health care is often not determined entirely by individual choice, but rather by the wealth of a country, its commitment to population health, and the distribution of its resources. When one includes this understanding of the economic context that dictates individual access to health care, context itself seems like a grossly unfair justification for violating the principle of beneficence. Here, the 'standard of care' argument only serves to exploit the individual, and take advantage of her circumstance and poverty.

Furthermore, we can expand the scope of context to include both an evaluation of a nation's internal health care priorities and overriding global economic inequities. Recognizing the role of developing nations in the global economy, we see that it is not entirely by choice that developing countries provide a standard level of care that does not include adequate MTCT prophylaxis. Developing countries are in fact given very little option under the continuing reverberations of the 1980s debt crisis. After heavily borrowing from the International Monetary Fund and the World Bank, countries have faced 'forced' economic reform through the structural adjustment policies of these lending institutions such as the devaluation of currency and enforced transition into export-based economies. This economic re-structuring has either required or caused a significant erosion of social service infrastructure, including health care and has particularly impacted many of the most vulnerable populations [9]. By dismissing the international forces that define the range of economic options available to developing countries, we isolate the problems of the developing world and allow ourselves to ignore our relative role and responsibility. Thus, within this broader scope of context, developed nations leading the research in question should recognize their interconnected role in the determinants of global inequity and should caution their support for the 'standard of care' argument which only serves to reinforce such inequity.

When expanding the parameters of context, the use of placebo-controlled trials in developing countries also fails to meet the principle of justice. This principle ensures that those who bear the burden of research risk will ultimately receive the benefits of the research [10]. In other words, specific populations should never be targeted due to their availability or compromised position. In the early debate over the short-course AZT trials, Satcher and Varmus argued that this principle was being satisfied since the trials were specifically investigating cost effective treatments for the HIV epidemic that was disproportionately affecting the world's poorest nations [11]. This argument hinges on a definition of context that isolates developing countries as independently dealing with an overwhelming epidemic and obscures our ability to apply the principle of justice at an appropriate level of analysis.

To assess the principle of justice within a broader understanding of the HIV epidemic, we must determine whether the research subjects would indeed receive adequate benefits for their participation. At the beginning of the debate, Annas and Grodin challenged the notion that an affordable treatment would ever be operational given the exceedingly low health care resources available to developing countries [12]. While the feasibility of implementation may have been questionable in 1998, there has been a global commitment to the prevention of MTCT and some notable successes in reducing the rate of MTCT with short course ARV regimens. Unfortunately, programmes designed for resource poor areas continue to fall short of the overwhelming success seen in developed countries where studies with highly active antiretroviral therapy demonstrate transmission rates of 1% in a non-breastfeeding population [13]. In contrast, the World Health Organization estimates that in developing countries, only 1% to 35% of populations have access to preventative care, with the lowest coverage in countries most significantly affected by the HIV epidemic [14]. Also, in 2002, it was estimated that 800 000 children acquired HIV infections, the vast majority of which were in the developing world [15]. While resource-rich populations are virtually beating vertical transmission, developing nations continue to struggle.

In terms of weighing the research subject's contributions to the trials against their benefits, which ten years later seems partial at best, an expanded context forces us to look at all the potential beneficiaries of this research. It has been argued that advances in more effective and cheaper methods of preventing vertical transmission are as likely to be implemented in developed countries as in developing countries [16]. The fact that these short-course regimens have not become standard in the developed world is due to their sub-standard reduction of transmission in comparison to more complex ARV regimens [17]. However, knowledge generated by these short-course trials has been applied to other research. The promising effects of short-course nevirapine trials in developing countries prompted researchers to study whether adding nevirapine to the more complex gold standard ARV regimen would further reduce transmission rates in the developed world [18]. This demonstrates that knowledge does not stay within segregated developing-developed contexts. Isolating the HIV epidemic as a developing world problem does not adequately acknowledge the global scope of the disease nor the subsequent global benefit of advances in treatment. Given the limited benefits to date of MTCT programs and this larger web of beneficiaries, it seems that while perhaps unintentional, the structure of current research serves to exploit impoverished populations for the benefit of science and more developed nations. In this context, the argument for the placebo-control trials violates the principle of justice.

## Where does the debate stand now?

The 'standard of care' debate has continued since the MTCT prevention trials and has prompted the inclusion of paragraph 29 in the Declaration of Helsinki:

the benefits, risks, burdens, and effectiveness of a new method should be tested against those of the best current prophylactic, diagnostic, and therapeutic method. This does not exclude the use of placebo, or no treatment, in studies where no proven prophylactic diagnostic or therapeutic method exists [19].

This attempt to create more stringent standards for the use of placebo-controlled trials regardless of the contextual standard of care, has been attacked by the international community as "out of touch with contemporary thinking" [20] and overly constitutional [21]. In response to these changes, there has been a flurry of independent international organizations writing their own, opposing ethical standards. Many, such as the Nuffield Council on Bioethics and the Council for International Organizations of Medical Sciences, have come to the conclusion that there should not be an absolute ruling on this matter, but that study design can be qualified when all other standards are satisfied and a sound scientific reason can be given for using a placebo-control arm [22]. This is treading on dangerous territory as the demands of science may outweigh ethical safeguards for individuals. It seems the debate has landed us back at the beginning with the Nuffield Council's new guidelines:

Wherever appropriate, participants in the control group should be offered a universal standard of care for the disease being studied. Where it is not appropriate to offer a universal standard of care, the minimum standard of care that should be offered to the control group is the best intervention available for that disease as part of the national public health system [23].

What determines the appropriateness of offering a universal standard of care may be scientific criteria or, as Schuklenk argues, may be erroneously conflated with economic criteria such as the low standard of care available in resource-poor settings [24]. This dangerous return to justifying a double standard for research in developing countries shows us that instead of building a mature consensus around the application of research ethics to developing countries, the discourse has become even further ensconced in the isolating and narrow context of the developing world.

## Conclusion

The arguments put forward to understand the ethical dilemma created by the short-course ARV trials for the prevention of MTCT of HIV should not be interpreted to mean that research should never be done in developing countries. Rather, developed nations need to honestly assess their role in such research, take responsibility for their actions, and abstain from the exploitation of ethical loopholes as provided by the contextual nature of bioethics. It is essential that we consider context in our ethical deliberations, but we must be critical of our definition of context. It would be tragic if we allowed ethical principles to be manipulated for the exploitation of vulnerable populations, the psychological comfort of the true beneficiaries, and the effacement of real differences between individuals and populations. To this end, we must always remember that the inclusion of context is a corrective to traditional ethics, not an invitation to exploitation. As demonstrated above, the framework of the short-course ARV trials is fundamentally challenged when context is taken seriously. However, the current global situation does not engender optimism that this exploitative research constitutes an isolated incident. We must not shirk our own recognized ethical responsibilities – at the heart of research design we must situate the proper articulation of context, towards which I submit the above as a first step.

## Abbreviations

**AIDS **Acquired Immune Deficiency Syndrome

**ARV **Antiretroviral

**AZT **Zidovudine

**CDC **Center for Disease Control

**HIV **Human Immunodeficiency Virus

**NIH **National Institutes of Health

**MTCT **Mother to child transmission
